# MIC_Locator: a novel image-based protein subcellular location multi-label prediction model based on multi-scale monogenic signal representation and intensity encoding strategy

**DOI:** 10.1186/s12859-019-3136-3

**Published:** 2019-10-26

**Authors:** Fan Yang, Yang Liu, Yanbin Wang, Zhijian Yin, Zhen Yang

**Affiliations:** 1 School of Communications and Electronics, Jiangxi Science & Technology Normal University, Nanchang, 330003 China; 2 Department of Biological Chemistry and Molecular Pharmacology, Harvard Medical School, Boston, MA 02115 USA

**Keywords:** Bioimage informatics, Protein subcellular localization, Frequency domain feature, Monogenic signal, Image intensity encoding strategy, Multi-label classifier chain

## Abstract

**Background:**

Protein subcellular localization plays a crucial role in understanding cell function. Proteins need to be in the right place at the right time, and combine with the corresponding molecules to fulfill their functions. Furthermore, prediction of protein subcellular location not only should be a guiding role in drug design and development due to potential molecular targets but also be an essential role in genome annotation. Taking the current status of image-based protein subcellular localization as an example, there are three common drawbacks, i.e., obsolete datasets without updating label information, stereotypical feature descriptor on spatial domain or grey level, and single-function prediction algorithm’s limited capacity of handling single-label database.

**Results:**

In this paper, a novel human protein subcellular localization prediction model MIC_Locator is proposed. Firstly, the latest datasets are collected and collated as our benchmark dataset instead of obsolete data while training prediction model. Secondly, Fourier transformation, Riesz transformation, Log-Gabor filter and intensity coding strategy are employed to obtain frequency feature based on three components of monogenic signal with different frequency scales. Thirdly, a chained prediction model is proposed to handle multi-label instead of single-label datasets. The experiment results showed that the MIC_Locator can achieve 60.56% subset accuracy and outperform the existing majority of prediction models, and the frequency feature and intensity coding strategy can be conducive to improving the classification accuracy.

**Conclusions:**

Our results demonstrate that the frequency feature is more beneficial for improving the performance of model compared to features extracted from spatial domain, and the MIC_Locator proposed in this paper can speed up validation of protein annotation, knowledge of protein function and proteomics research.

## Background

Human protein subcellular localization prediction is an important component of bioinformatics. Identifying the subcellular locations of proteins can improve our understanding of their functions, mechanisms of molecular interaction, genome annotation and identification of drug targets [1, 2]. For example, protein synthesized from ribosome must be transported to their corresponding subcellular locations to fulfill their functions. Aberrant subcellular localization of protein can lead to serious loss of biological function or disorder occurrence in organisms and can even cause cancer [3]. Diabetes, blindness and certain forms of cancer have been demonstrated to be caused by the malfunction of G Protein-Coupled Receptor (GPCR) signaling pathways [4, 5]. Moreover, understanding of protein subcellular localization can greatly improve target identification during drug discovery. In the case of membrane proteins and secreted proteins, they are easily accessible by drug molecules due to their localization in the cell membrane or on the cell surface. It is well known that the traditional protein subcellular location annotation is derived from biological experiments in wet laboratory, however, computational models offer an attractive complement to time-consuming and laborious experimental methods [6, 7].

Currently, a large number of automated prediction models have been developed for correctly predicting the subcellular locations of protein [8–10]. These prediction models can be divided into two categories in terms of processing target datasets, i.e., sequence-based [11–14], which uses the amino acids sequence as the input protein information, and image-based [15–18], which employs the biology image as the target dataset.

Efforts on sequence-based protein subcellular localization have been made by many research groups, such as Chou group, Briesemeister group, Wan group and Almagro group, and the corresponding software is Cell-Ploc, YLoc, iLoc-Hum, FUEL-mLoc, SpaPredictor and DeepLoc [19–24]. For instance, Chou et al. proposed a high-performance prediction model, iLoc-Hum, which can handle proteins with single-labeled and multi-labeled subcellular locations [20]. By applying the gene ontology (GO) and position specific scoring matrix (PSSM) sequence information and K-nearest neighbor classifier (KNN) classification, iLoc-Hum achieve a remarkably higher success rate at 76%, and a user-friendly web-server is developed. FUEL_mLoc is proposed to predict with single- or multi-label, and it uses the key go terms to analyze how a prediction is made and it can predict several species. The experimental results proved that FUEL-mLoc outperforms state-of-the-art subcellular localization predictors [22]. However, with the technology development in gene sequencing, the imperfection of protein sequence annotation was preferred by scientists [25, 26]. Then several genes sequencing reannotation tools are designed for checking and correcting the error of annotation. They encouraged researchers to realize that these sequence-based methods may not be significantly reliable [27].

Moreover, the sequence-based methods are not sensitive to protein translocations, especially when dealing with cancer. In detail, human health is reflected by cells, which are restricted by the internal ecological environment of human body. When unavoidable changes of environment occur, cells must have complex collaborative response, i.e., protein translocation [14]. Amino acid sequence itself does not change when the protein trans-location in cancer cell environment. Hence, image-based protein subcellular localization prediction models have gradually become a research hotspot [28–30]. Murphy group proposed a framework for the construction of image-based protein subcellular localization prediction, and the prediction framework was first applied to the Human Protein Atlas (HPA) database [16]. This initiative is regarded as the pioneering work in the field of image-based subcellular localization prediction.

In the following years, an increasing number of image-based protein subcellular localization prediction models have been proposed based on the combination of image processing technologies and machine learning algorithms. For example, Boland et al. utilized the back-propagation neural network classifier and subcellular location features (SLFs) to recognize the subcellular localization of Hela cells [31], however, the local information of sample was not revealed. Muhammad Tahir et al. proposed the SVM-SubLoc method, which focuses on the combination of the Haralick feature and local image descriptor, then feeds into the support vector machine (SVM) classification. The SVM-SubLoc model can achieve 99.7% prediction accuracy in Hela cells dataset [32]. Lin group proposed a new learning algorithm named AdaBoost.ERC. They utilized the error-correcting output codes (ECOC) coding strategy and the boosting method to improve the prediction accuracy [33]. Although the model mentioned above can obtain high accuracy, the involved features are extracted in spatial domain, which may be attributed to the limited image processing technology.

To describe local features more accurately, XU et al. first proposed the local binary pattern (LBP), a popular local image descriptor applied in the field of image retrieval, to protein subcellular images. Experimental results showed that LBP plays a significant role in improving the performance of prediction model by capturing the texture information of immunohistochemistry (IHC) images [17]. Coelhp L P et al. obtain the interest regions of IHC image by using the K-means method within the target image [18]. The feature descriptor is calculated in the interested regions of image. These entirely featured descriptors generated the local feature by clustering method. Although the approach achieved an improvement in the classification accuracy, the number of K-means clustering centers may cause fluctuations in the performance of prediction model for various datasets. For instance, the method just achieves 78.9% classification accuracy in the HPA dataset [34]; in contrast, 94.4% classification was obtained in the Hela2D dataset [35]. Shao group made efforts on the improvement of accuracy by using a novel voting strategy in decision level and taking the different relationship of labels into account. Although the method achieved high prediction accuracy, it was unable to handle multi-label protein subcellular location prediction [15]. Jieyue L and Newberg J et al. proposed to update the subcellular localization annotation of datasets by using the hierarchical clustering method and SVM classification, followed by continuously revising the subcellular localizations of test samples. Godinez W J et al. proposed M-CNN prediction model, which uses the convolution neural network (CNN) with multi-scale architecture, to predict image subcellular localization in eight published datasets. Although the experimental result showed that M-CNN achieved around 95% prediction accuracy in the seven datasets more than these popular network architectures, such as AlexNet and GoogleNet [36–38], M-CNN merely obtained the 77% prediction accuracy in the HPA dataset, as the HPA dataset consists of image with multi-label.

Moreover, many efforts have been made on the algorithm level [39–41]. Wei group proposed a novel feature selection method that used the biology background to set up a regularization item so as to optimize the feature selection method, and this method can select more informative feature subsets [40]. The Sullivan group innovatively used the online game (EVE Online) to attract the numerous participants to annotate the subcellular locations of protein image based on both of the transfer learning framework and the deep learning method to build the automated Localization Cellular Annotation Tool (Loc-CAT). This work not only achieved the F1 score of 0.74 but also proposed a novel approach to obtain the precious annotated data by the online game [41].

The contributions made by the predecessors in the field of protein subcellular localization prediction, especially in imaged-based, should be positively evaluated, however, three shortcomings can be summarized as follows.

Firstly, the labels of benchmark dataset in published works have been updated by database, such as HPA. Although the prediction accuracy at that time was quite gratifying, it would greatly reduce the credibility of the prediction model if the training samples used in the prediction model construction are involved in the label updating of database. Obviously, it is meaningless to accurately predict an error or a failed label, and the corresponding training samples can also be treated as obsolete data. Different from face and natural images, the label information of protein image datasets is updated regularly to ensure that the subcellular location corresponding to a sample image is true and accurate. For instance, the subcellular location of gene “ENSG00000182606” is reported “Cytopl” in [17], while the subcellular location of gene is updated “ER” and “Nucleoplasm” in version 18 of HPA database. The label of “ENSG00000155876” in HPA has been updated to Golgi apparatus and Vesicles in the latest version while its labels reported in [17] are “Golgi apparatus”, “Lysosomes” and “Vesicles”. Inspired by this, the latest datasets from HPA have been collected and collated as our benchmark instead of obsolete data.

Secondly, they lack of in-depth understanding of protein image signals. For a target protein image, it is not just a digital image, but more importantly, it is still a 2-dimension signal, which is often overlooked. Researchers are more eager to find a simple image descriptor to extract features from protein images rather than taking the time to figure out the 2-dimension signal. For example, LBP and its variation, local ternary pattern (LTP) and local quinary pattern (LQP), are employed to extract local feature of protein IHC images [42, 35]. These kinds of image descriptors focus on encoding the gray level information of image in spatial domain rather than considering other aspects of image, such as the local energy, structure and geometry information, which can be obtained from the transformation or frequency domain of image signal [43]. Even for complicated feature descriptors, such as completed local binary pattern (CLBP) and local tetra pattern (LTrP), can capture more local information [44, 45]; however, the target protein image is still encoded in grey level or spatial domain. This kind of roughly transplanted approach has ignored the biological properties of IHC protein images, which included multiple cells and can be sparse representation in frequency domain. Few researchers have been taking this point into account.

In this paper, to generally capture the essential local property of IHC image, Fourier transformation, Riesz transformation, Log-Gabor filter and intensity coding strategy are employed to obtain frequency feature based on three components of monogenic signal with several frequency scales. 2-dimension fast Fourier transform is employed to convert target protein channel from spatial domain into the frequency domain, and then the Riesz transformation [46] is employed to obtain two frequency responses in orthogonal directions [47]. To improve the robustness of model, the convolution of three parts, i.e., original frequency information and two frequency responses of Riesz transform, and Log-Gabor band-pass filter with different frequency scales is calculated. It is known that the detail information of IHC image, e.g., slight textures and edges, mainly concentrated on the high frequency band. In addition, larger frequency response can be obtained, if the frequency of local texture information is closer to the center frequency of Log-Gabor filter, and vice versa. The inverse 2-dimension fast Fourier transform converts three parts into the spatial domain, and the monogenic signal of image can be represented. By using various mathematical formulas, the three components of monogenic signal of protein channel can be calculated, namely, local amplitude, phase and orientation (APO). These three components denote to the energetic, structural, and geometric information of target protein image, respectively. The details for corresponding encoding strategies ara given in the following section.

Thirdly, it is well-known that above 50% of proteins are found in two or more subcellular locations. An effective and accurate prediction model should be capable of handling multi-label datasets, and it is critical to capture the dynamic transfer of proteins between different subcellular locations and to screen for cancer biomarkers. Xu et al. proposed an image-based multi-label protein subcellular prediction model CorrASemiB based on the combination of Bayesian theory and variety decision strategies [48]. The CorrASemiB employed the binary relevance (BR) classification as the multi-label classification, which leads the neglect of the correlation of subcellular localizations. In order to find the correlation between different subcellular locations, Wang group proposed the random label selection (RALS) to more accurately predict the subcellular localizations of protein with multi-label, which learned the correlation of different subcellular localizations from datasets by randomly selected labels as the additional features adding into the original feature space [49]. However, the randomly selected labels will lead to the prediction performance instability of model. Zhou et al. used the multi-view complementary protein information, i.e. GO, conserved domain database (CDD) and amino acid composition (AAC), to build the prediction model [9]. While this method achieved an increase in the prediction accuracy at 5–11% because the sample feature was extracted from the multi-view of protein, the correlation of labels and the hierarchical structure of GO terms are ignored.

Considering the importance of multi-labeled proteins, the predictive model is expected to handle multi-labeled datasets, a chained classification is proposed in this paper. The experimental results show that the subset accuracy of the proposed prediction model can achieve 60.56% classification accuracy and outperform the existing prediction models.

## Results

The 5-fold cross-validation is utilized to split the train set and test set on the benchmark dataset in this paper. The benchmark dataset consists of 3240 IHC images, and the proportion of image with multi-label is 25%, i.e., 824 multi-label IHC images in total. The numbers of subcellular locations involved in benchmark are seven, i.e., “Cytosol”, “Endoplasmic reticulum”, “Golgi apparatus”, “Nucleoli”, “Mitochondria”, “Nucleus” and “Vesicles”. A total of 1864-dimension features, derived from SLFs and frequency feature, have fed into subsequent classifier chains (CC). In the next section, the MIC_Locator^X_S^ (X is one of A, P and O components; S represents the scale factor from 1 to 5) prediction model is trained by the combination of global features and local image descriptor with different frequency scales in these components of monogenic signal. The MIC_Locator^X_E^ prediction model (X is A, P and O components) denotes to the ensemble prediction model of three APO components. These weighted ensemble methods are used to fuse all single prediction models for constructing the prediction model MIC_Locator.

### The performance of MIC_Locator with frequency feature on new benchmark dataset

**Fig. 1:**
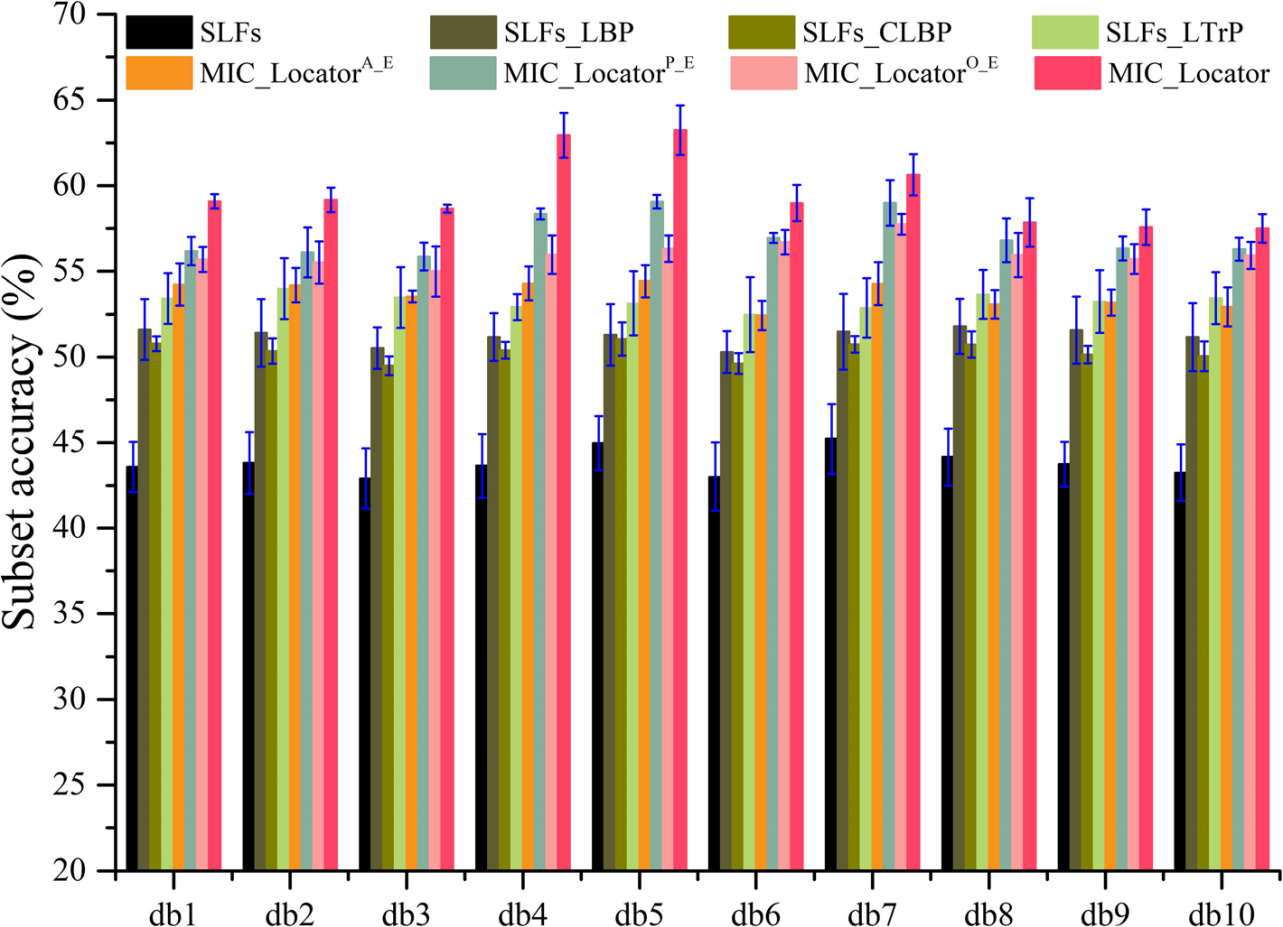
The classification results of prediction model trained with the combination of global feature and different local feature based on the 5 times 5-fold cross-validation, and the corresponding mean and standard deviation of each case are also given

In this section, we aim to compare the performance of frequency feature with different local image descriptors, namely LBP, CLBP and LTrP. The SLFs feature with 10 dbs, which derives from the 10 vanishing moments of 2-dimension wavelet analysis function, e.g. db1-db10, is directly combined with these different local image descriptors and frequency domain feature as the sample feature. As the results (mean and standard deviations) are shown in Fig. 1, there are two distinct trends. One is that the MIC_Locator achieves the best classification accuracy, and the other is that the ensemble prediction model of APO components is more high-performance than these local image descriptors extracted from spatial domain.

**Fig. 2:**
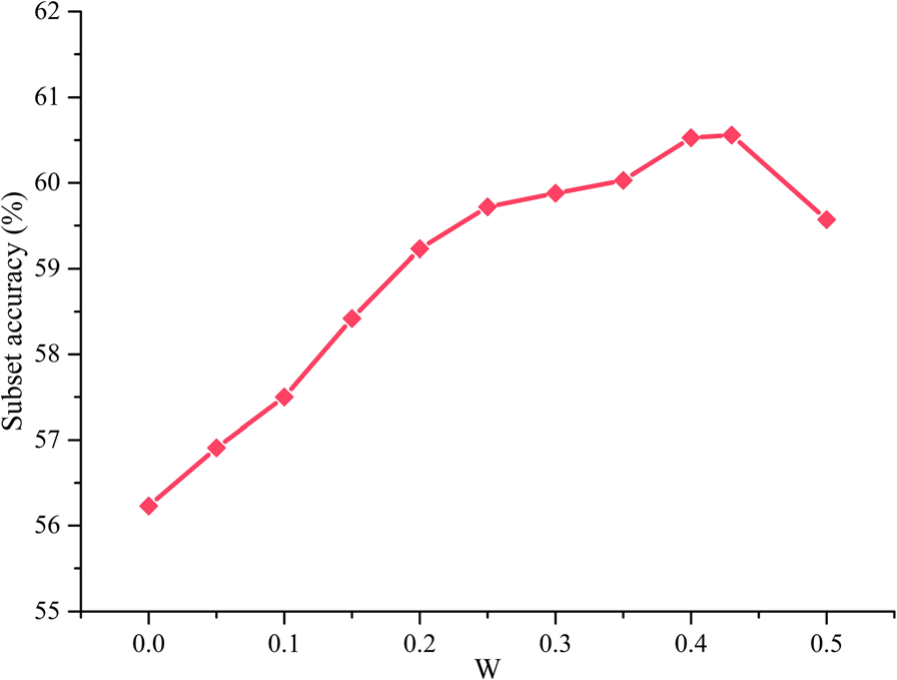
The subset accuracy of MIC_Locator fluctuates with the weighted parameter *W*

From Fig. 1, the MIC_Locator can achieve the 63.24% subset accuracy in db5, but the classification SLFs_LBP, SLFs_CLBP, SLFs_LTrP just achieve lower accuracy at 51.29, 51.05 and 53.13%. Consistent with the above conclusion, MIC_Locator achieves the best performance in other dbs. The ensemble prediction models of APO components are fused by the weighted ensemble algorithm. The weight parameter of weighted ensemble method is obtained by the grid research from 0.1 to 0.5 with the step of 0.01 based on db4, and the producer of experiment has been shown in Fig. 2. The weight parameter is set to be 0.43 as the final weight parameter, when the MIC_Locator achieves the highest subset accuracy.

An expected result is observed that the ensemble prediction model MIC_Locator^X_E^ can extremely improve the classification accuracy of prediction model except the MIC_Locator^A_E^. For instance, MIC_Locator^P_E^ and MIC_Locator^O_E^ respectively achieve 59.06 and 56.31% subset accuracy, which exceed the SLFs_LBP to 7.77 and 5.02% in db5. Nevertheless, there is a deficiency that MIC_Locator^A_E^ achieves relatively low classification accuracy, since the ability A component to describe subtle texture information is poor compared with P and O components. This result can be attributed to the fact that the slight texture information is more sparely expressed in the frequency domain making it easily to be captured by the PO components, and then MIC_Locator^P_E^ and MIC_Locator^O_E^ can be superior to SLFs_LBP. The above-mentioned reasons can be validated with experimental results in the next section.

Furthermore, in the comparison of local image descriptors extracted in the spatial domain, the LTrP achieve the highest classification accuracy than the LBP, CLBP. Specifically, SLFs_LTrP prediction model trained by the combination of SLFs and LTrP local image descriptor can achieve 53.13% subset accuracy in db5. The results demonstrated that the LTrP local image descriptor can preferably extract the texture information of image, as the LTrP captures the statistic information of image by comparing the consistency of center pixel with neighboring pixels. Although the LTrP used a more complex local image descriptor coding strategy, higher subset accuracy is achieved by the MIC_Locator at 63.24% in db5 as the local image descriptor of MIC_Locator codes the frequency information rather than the spatial information. The classification accuracy of prediction model SLFs_LBP achieves 51.29% subset accuracy in db5, which is 1.84% lower than the prediction model SLFs_LTrP. Because the definition of LBP is concerned the difference between the center pixel and its neighboring in gray level to capture the statistic information of image. The SLFs_CLBP prediction model achieves limited classification accuracy at 51.05% in db5. The reason is that the CLBP local image descriptor compares the gray level of center pixel with the average gray level of whole image to add center pixel information, which cannot more precisely capture the essential property of center pixel. In addition, while the local image descriptor as a complementary feature combined with the SLFs, the prediction model can hugely increase the classification accuracy. For example, the prediction model SLFs obtain the lowest classification accuracy in 44.97%, owing to the lack of local image descriptor. The SLFs_LTrP, SLFs_LBP, SLFs_CLBP prediction model respectively achieve a higher classification accuracy compared the SLFs prediction to 8.19, 6.29 and 6.08% in db5. Although the performance of local image descriptors extracted from the spatial domain has been validated, it is still inferior to MIC_Locator. Hence, we have made further analysis to verify and reveal the internal logic, such as the analysis of Log-Gabor filter, coding strategy, APO components and multi-scale.

### Performance of log-Gabor, image intensity coding strategy and classifier chain

In this section, to validate the advantages of parts, namely Log-Gabor filter, image intensity encoding strategy and CC, we respectively compare the MIC-Locator and the MIC-Locator without each part.

**Fig. 3:**
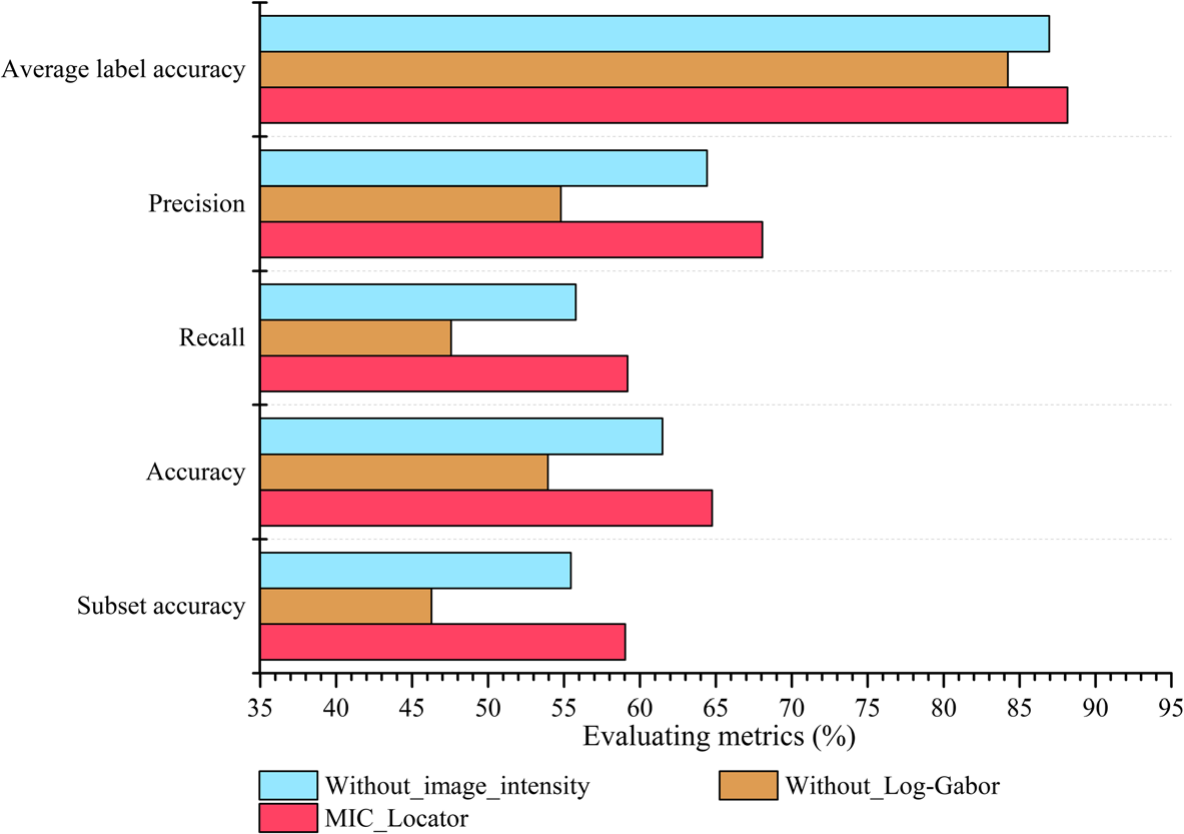
The results of various evaluation metrics for MIC_Locator, Without_image_intensity and Without_Log-Gabor on db3

The constructed MIC_Locator prediction model without Log-Gabor filter and image intensity encoding strategy is named as Without_image_intensity and Without_Log-Gabor. As shown in Fig. 3, the experimental results illustrate that the MIC_Locator without the Log-Gabor and image intensity coding strategy achieve lower performance. Specifically, the MIC_Locator achieve 59.04% subset accuracy in db3, but the Without_Log-Gabor and Without_image_intensity just obtain 46.28 and 55.46%. We can draw a conclusion that the Log-Gabor filter and image intensity coding strategy actually play an indispensable role in contributing the performance of MIC_Locator.

Furthermore, the CC classification is replaced by the BR multi-label classifier. The Table 1 investigates that the performance of MIC_Locator based on the CC and BR in 10 dbs in terms of overall, single-labeled and multi-labeled subset accuracy. As can be seen, the CC outperforms BR in the MIC_Locator^A_E^, MIC_Locator^P_E^ and MIC_Locator^O_E^ in all evaluation indexes. Although the MIC_Locator with BR classifier slightly outperforms the CC classifier at 0.75% in terms of overall subset accuracy, the CC can extremely boost the multi-labeled subset accuracy from 19.96 to 31.30%. Considering the CC is importantly effective for determining subcellular localization of multi-label proteins. Hence, the CC and frequency feature are jointly leveraged to constructing the MIC_Locator.

**Table 1:** The comparison of subset accuracy on both overall, single-label and multi-label testing dataset of MIC_Locator by using BR and CC in 1–10 dbs

	CC			BR		
	Subset accuracy	Single-labeled subset accuracy	Multi-labeled subset accuracy	Subset accuracy	Single-labeled subset accuracy	Multi-labeled subset accuracy
MIC_Locator^A_E^	54.73%	64.26%	26.90%	51.14%	63.47%	15.26%
MIC_Locator^P_E^	58.08%	67.45%	30.70%	55.25%	67.33%	20.06%
MIC_Locator^O_E^	57.71%	67.36%	29.52%	56.12%	68.80%	19.18%
MIC_Locator	59.86%	69.63%	31.30%	60.61%	74.56%	19.96%

### Results of exploration of the three components from monogenic signal

**Fig. 4:**
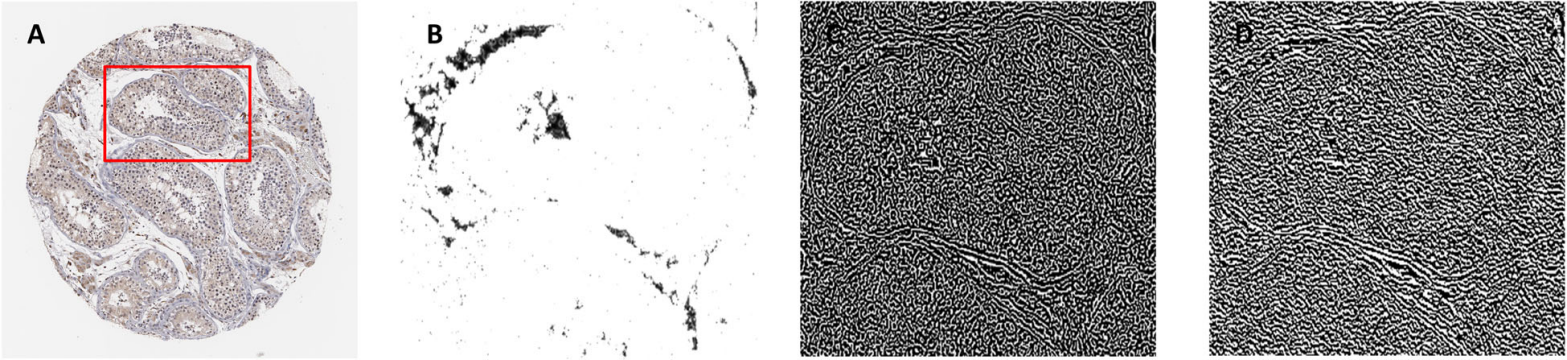
The comparison of ability in capturing slight texture feature on these APO components of image based on a given local patch in an IHC image. **a** Denotes to an IHC image derived from the “ENSG00000013364” and the corresponding subcellular location is “Cytosol”. An example of local patch region is presented in the original IHC image by marking red rectangle. The APO components on this local patch are separated in frequency domain and inverse transform (Fourier Inversion) to spatial domain for easy visualization. **b** Denotes to amplitude component under the local patch. **c** Represents the phase component under the local patch. **d** Represents the orientation component under the local patch

An obvious conclusion can be drawn from Fig. 1 that frequency features are more discriminative than SLFs and the original spatial feature, and can greatly improve the accuracy of the prediction model; however, we are more interested in which component plays a more important role in the whole frequency domain. Hence, the APO components are visualized and showed intuitively in Fig. 4.

It is well known that the phase spectrum is most important in frequency domain analysis of the signal, and the consistent conclusion can be observed in Fig. 4. Firstly, an IHC image is selected from the benchmark datasets, and the selected patch is marked by the red rectangle frame. Secondly, the local patch in these three components is commonly amplified, which are shown in Fig. 4. It is clear that the amplitude component mainly reflects the outline of image in local patch, and the phase component extremely reflects the slight texture, and the orientation component presents the texture information along the gradient direction.

**Fig. 5:**
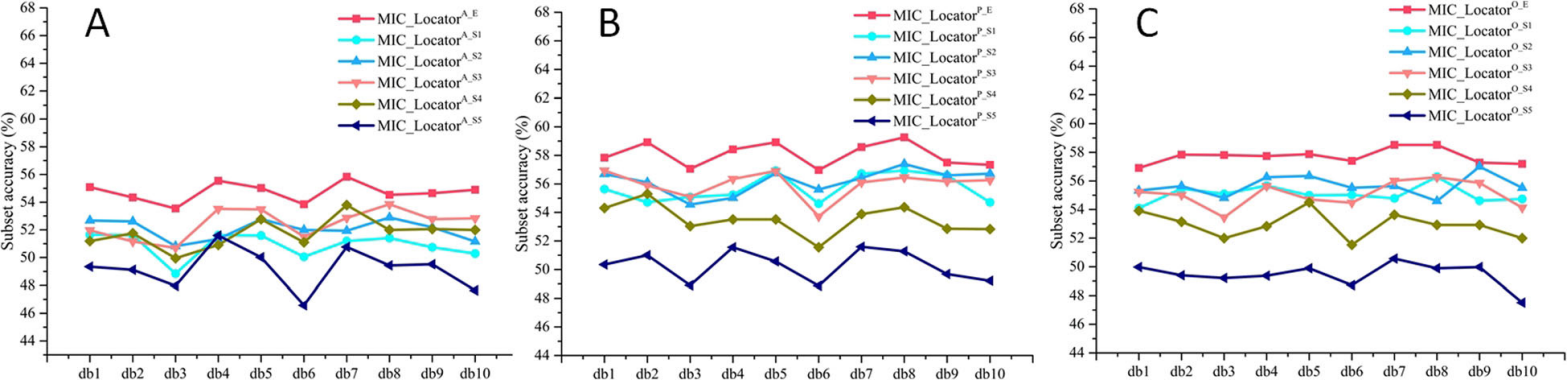
Compared the ensemble prediction model with each single prediction model based on the APO components, local amplitude, local phase and local orientation. **a** Compares MIC_Locator^A_E^ with MIC_Locator^A_S1^ to MIC_Locator^A_S5^ based on 10 dbs. **b** Compares MIC_Locator^P_E^ with MIC_Locator^P_S1^ to MIC_Locator^P_S5^ based on 10 dbs. **c** Compares MIC_Locator^O_E^ with MIC_Locator^O_S1^ to MIC_Locator^O_S5^ based on 10 dbs

Another important finding was that the phase component captures more frequency information than other components. Specifically, the orientation component vaguely presents the outline of local patch in the upper right of Fig. 4d, but the phase component more distinctly presents the texture of local patch in the upper right of Fig. 4c. In order to verify the conclusion of the subjective evaluation, some essential experiments are carried out and the corresponding results are shown in Fig. 5. The result of FSL_PSL^P_E^ outperforms phase component can significantly reflect frequency information.

### Results of MIC_Locator on different frequency scales

To gain better understanding of which frequency scale is better and whether fusing these prediction model with single frequency scale can obtain more benefits, the performance of MIC_Locator with different frequency scales on APO components are compared, and it is necessary for us to verify whether the conclusion mentioned above is consistent at all scales.

In this section, the scale index is set from 1 to 5, which affects the center frequency that makes the Log-Gabor band-pass filter has different frequency responses, and the results are showed in Fig. 5. The prediction model with frequency scale from 1 to 3 can achieve superior classification performance. For instance, the MIC_Locator^P_S3^ achieves 55.89% classification accuracy in db2, while the MIC_Locator^P_S4^ and MIC_Locator^P_S5^ respectively achieve 55.3 and 51% classification accuracy; the MIC_Locator^O_S3^ achieve 55.02% classification accuracy in db2, whereas the MIC_Locator^O_S4^ and MIC_Locator^O_S5^ respectively achieve 53.14 and 49.4% classification accuracy.

Furthermore, these ensemble prediction models of each component, MIC_Locator^A_E^, MIC_Locator^P_E^ and MIC_Locator ^O_E^, achieve the highest prediction accuracy on each db. For example, MIC_Locator^P_E^ achieves the 58.92% classification accuracy, while the MIC_Locator^P_S1^ and the MIC_Locator^P_S5^ respectively achieve 56.94 and 50.57% classification accuracy in db5, since these ensemble prediction models fuse the advantage of each single prediction model. From the Table 1, the ensemble prediction model of phase components MIC_Locator^P_E^ achieve the highest subset accuracy than MIC_Locator^A_E^ and MIC_Locator^O_E^ on 10 dbs by 3.35 and 0.37%, as the phase component is preferable to capture the texture information of image; the MIC_Locator, however, outperforms the MIC_Locator^P_E^.

### Performance validation of MIC_Locator on both single-label and multi-label datasets

In order to validate the performance of proposed prediction model MIC_Locator, we compare MIC_Locator with opened and popular methods in db4. The comparison experiments can be carried out divided into two parts, namely multi-label part and single-label part.

An excellent prediction model, accurate and efficient prediction of single-labeled samples in the benchmark dataset is the basic guarantee of the generalization ability of prediction model. The performance of MIC_Locator is compared with the [15, 16] in predicting the single-labeled sample part. The accuracy, recall and precision are used for the evaluation index, and the experimental result has been shown in Table 2.

**Table 2:** The performance comparisons of single-label prediction model on db4

Prediction model	Accuracy	Recall	Precision
Coelho et al. [16]	56.34%	54.35%	57.97%
Wei et al. [15]	60.46%	57.75%	66.84%
MIC_Locator	71.27%	70.54%	72.00%

The [16] uses the SLFs as the sample feature, and the linear SVM is applied as a classification to predict the subcellular location of test sample. The LBP and SLFs are combined as the sample features feeding the SVM, and the SC-PSorter voting strategy and multi-kernel learning method are used to enhance the performance of [15]. To obtain an objective comparison result, these single-labeled samples are selected from benchmark datasets as a dataset for the [15, 16], as the benchmark datasets include the multi-labeled protein, which disturbers the performance of single-labeled prediction model [15, 16]. Meanwhile, MIC_Locator only predicts the single-labeled sample in the benchmark dataset. Based on the 5-fold cross-validation, the MIC_Locator obtain 71.27% accuracy 70.54% recall and 72% precision, and these three metrics are higher the [15, 16]. The better performance of MIC_Locator mainly owes to the following two aspects: (i) we use the frequency feature of IHC to construct prediction model and (ii) fusing the single prediction based on several frequency scales enhances the robustness and general ability of MIC_Locator.

To further confirm the performance of MIC_Locator in multi-label part, the MIC_Locator is compared with the iLocator, which belongs to the multi-label subcellular localizations prediction model, and the experiment result is shown in Table 3. The accuracy, recall, precision and label average accuracy are used for the evaluation index, and these evaluation indexes are defined in [17, 61]. The better performance of MIC_Locator mainly owes to the following two aspects: (i) we use the frequency feature of IHC to construct prediction model and (ii) fusing the single prediction based on several frequency scales enhances the robustness and general ability of MIC_Locator.

**Table 3:** The performance comparisons of multi-label prediction model on db4

Prediction model	Subset accuracy	Recall	Precision	Average label accuracy
iLocator [17]	54.81%	53.63%	57.51%	84.90%
MIC_Locator	60.43%	60.12%	69.24%	88.43%

Based on the original benchmark dataset and 5-fold cross-validation, the MIC_Locator achieve 60.43% subset accuracy, and it exceeds the iLocator by 5.62%. For the analysis of experiment result, it is described in the discussion section.

### Extended exploration results of MIC_Locator

It is well known that target images with high quality dyeing properties and accurate label are less than 50% in HPA. Some semi-supervised learning models are proposed to select properly from medium quality dyeing images and participate in the training stage of the model in order to solve the shortage of high quality dyeing sample. However, such kind of approach must be fully confident in the robustness of the prediction model. In this section, we compare the model proposed in this paper with the existing semi-supervised model. The experimental results show that the proposed model is better than the semi-supervised model. Moreover, transform the proposed model into a semi-supervised model is a very interesting follow-up work.

In this section, we compared our prediction model with two popular semi-supervised prediction models, i.e. standard semi-supervised approach [39] and improved semi-supervised approach CorrASemiB [48]. The results of performance comparison have been shown in Table 4. Referring to the [39], this standard approach is to select properly based on the consistency between the prediction labels from the proposed supervised learning model and the true labels. As for CorrASemiB, integrating the different organelles correlation emerges a DAG structure by the Bayesian algorithm that each node represents a subcellular location, and the edge of DAG structure symbolizes the reliable relations between two subcellular locations.

**Table 4:** The subset accuracy (%) for the different prediction models based on 10 dbs

	db1	db2	db3	db4	db5	db6	db7	db8	db9	db10
Hady et al. [39]	36.37	36.37	35.93	36.49	37.55	35.75	37.45	37.45	36.93	37.80
CorrASemiB [48]	48.38	48.38	49.00	48.07	49.77	50.23	46.21	49.61	49.46	48.53
MIC_Locator	59.85	60.43	59.04	60.43	60.37	59.04	60.04	60.56	59.47	58.98

Two consistent conclusions can be observed from the comparison experimental results. Firstly, the MIC_Locator achieve the highest subset accuracy in 10 dbs, and the identical conclusions were obtained in the Fig. 1. Since we utilized amplitude, phase and orientation components in various frequency scales to describe the IHC image which can not only describe the energetic, structural, and geometric information of protein channel, but also the texture of protein channel with different frequency spans can be captured; Secondly, The performance of the standard semi-supervised [39] only can reach 36% subset accuracy on the new benchmark dataset while the result of improved semi-supervised approach is 12% higher than the standard approach. Refer to [39] approach, the BR classification is employed as multi-label classification which cannot consider the correlation between different subcellular locations leading lower classification accuracy. The CorrASemiB approach achieves progress in prediction performance compared to [39] approach, as the Bayesian network is applied to guide the constructing of model. However, the lack of efficient local image descriptor results in limited prediction accuracy.

## Discussion

By comparing local image descriptors deriving from spatial domain and frequency information, it is observed that several important factors contributed to the excellent performance of MIC_Locator. Firstly, extracting frequency features by three different aspects of image, namely APO components, is superior to capturing the texture information of image from the amplitude, phase and orientation perspective of image. Secondly, as shown in Fig. 1, fusing in decision level based on several single frequency scales and APO components not only can integrate the advantages of each prediction model but also can enable multiple prediction models to complement each other, and ultimately obtain better classification accuracy.

To get an inquiry of MIC_Locator in depth, the comparison experiment had been carried out to explore the performance contribution of Log-Gabor filter, image intensity coding strategy and CC parts on the final prediction. As shown in Fig. 2, our experiment results demonstrate that the MIC_Locator without these three parts achieve limited performance, and identical conclusions can be obtained. Firstly, the Log-Gabor with different frequency scales can capture more frequency information distributing in various frequency bands and avoid the disturbance of DC. Secondly, the image intensity encoding strategy more accurately describes the distribution of local signal, and it enhances the discrimination of MIC_Locator. Finally, CC can significantly improve the classification accuracy for multi-label by capturing the correlation of different subcellular location.

It is well known that phase is the position of a point in time (an instant) on a waveform cycle in the field of physics and mathematics, and also a typical feature in frequency domain. Hence, P component is given a higher expectation, which means it will have a better performance in MIC_Locator while comparing with A and O component. By analyzing the experiment result of MIC_Locator under various APO components with qualitative and quantitative approaches, it is found that the phase component is indeed more superior to improving the performance of classification than amplitude and orientation components and extracting the slight texture information of image, which further demonstrates that the phase component plays a significant role in capturing the frequency information of sample. Furthermore, comparing with state-of-the-art methods belonging to both single-labeled and multi-labeled methods, the proposed MIC_Locator outperforms other baseline approaches shown in Tables 2 and 3 in terms of different evaluation indexes, which demonstrate again the high-performance of MIC_Locator. The reasons are summarized as follows. Firstly, the fine-grain information of IHC is transformed into the spare information in frequency domain by the Riesz transform, Fourier transform and the Log-Gabor with the multi-scale frequency factor, which is conducive to capturing the information of IHC. Secondly, APO components enable IHC information to be captured more completely, because the APO components reflect the energy, structure and geometry information of IHC rather than the gray level information. Thirdly, the LBP and image intensity coding schedules are commonly used to capture the statistic information of APO components. Finally, the CC classification is used to handle multi-label task, which considers the correlation of several subcellular localizations in the process of constructing prediction model. The result validates the advantage of MIC_Locator for the subcellular localization prediction of multi-label protein.

Owing to the advantage of semi-supervised model is that more training samples are used to enhance the generalization ability of the model in the training stage, two excellent semi-supervised models are proposed [39, 48]. Hence, the investigation on the performance comparison between MIC_Locator and some semi-supervised models had been carried out. As can be seen from the comparison results in Table 4, the proposed MIC_Locator is about 12% higher than the overall accuracy of the semi-supervised learning model. This is not to say that the semi-supervised learning framework does not work, but because semi-supervised learning is based on supervised learning. Once the quantitative features are weakly discriminative or the machine learning algorithms are not robust, and then the advantages of semi-supervised learning are difficult to fully exploit. Although MIC_Locator has a good predictive performance, more samples to participate in training are expected. However, it is an indisputable fact that high quality dyeing images are a minority in HPA database. Therefore, it is meaningful for MIC_Locator to combine with semi-supervised framework, and two advantages can be summarized as follows. Firstly, MIC_Locator achieved significant improvement can provide a very accurate and efficient supervised-prediction-model guarantee for the semi-supervised learning framework. Secondly, more medium quality dyeing images can make feature capture more comprehensive and accurate in frequency domain.

Furthermore, research work based on image signals is still very few while comparing with the study of protein subcellular localization prediction at the sequence level; however, the prediction model based on image signal of analysis is more visualized and interpretable, such as phase components shown in Fig. 4. Therefore, we believe that the combination of prior knowledge of protein at the sequence level and analysis at the protein robustness and generalization ability of the predictive model, which is also a very meaningful follow-up research direction.

## Conclusion

In this study, an accurate and effective multi-label protein subcellular locations prediction model named MIC_Locator is proposed. Experimental results have demonstrated that MIC_Locator can achieve 60.56% subset accuracy on the new multi-label benchmark dataset derived from version 18 of HPA. Different from the reported prediction model, MIC_Locator transforms IHC images into frequency domain to capture more discriminative information, i.e., amplitude, phase and orientation information. In detail, the frequency feature is extracted from the monogenic signal of image based on the different frequency scales. In addition, intensity encoding strategy is employed to provide complementary information. Finally Classifier Chain enables MIC_Locator to enhance the capabilities of handling the multi-labeled dataset efficiently.

In order to evaluate the overall capabilities of the proposed MIC_Locator model objectively, we analyzed the MIC_Locator model from multiple angles: Firstly, integrity evaluation of predictive models under the introduction of frequency domain features and classifier chain architecture in 10 dbs. The proposed MIC_Locator outperformed any other approaches in Fig. 1. Secondly, independent exploration in-depth of APO components to demonstrated that the P component outperforms A and O components in discriminative ability of prediction model. The relevant experimental results further validate our expectation that the phase information should have a more general meaning in the frequency domain signal; thirdly, study in-depth of the impact of different frequency scales and components on the prediction model, and the decision fusion also considered. Finally, based on all previous results mentioned above, the expanded experiment of the comparison between MIC_Locator and semi-supervised framework was carried out. This is because the high quality dyeing image samples are really limited in the HPA database, and we hope to further improve MIC_Locator. The experimental results show that the combination with the semi-supervised framework is indeed very sensible. Furthermore, we have made efforts on applying CNN into determining subcellular location. Due to the huge loss of gradient information in the high layer of CNN model, it remains a challenge for training a high-performance CNN model. In future work, we plan to develop a CNN model based on the residual network architecture so that the problem of gradient disappearance can be effectively solved.

From the perspective of model application, MIC_Locator can be used to automate annotation of proteins subcellular location, and contribute to revealing protein function. Moreover, the MIC_Locator can provide reliable indication of whether a certain protein is suitable as a cancer biomarker by capturing the transfer among its subcellular locations. Some initial results have been achieved but not reported in this paper.

## Methods

### Benchmark datasets

When it comes to image databases, HPA is undoubtedly one of the most popular protein image data sources in the world in recent years [2, 51–53]. It is a completely open database that allows academics and industry researchers to freely access to explore all human science issues related to human proteomics. The HPA project originated in 2003 is supported by the Knut and Alice Wallenberg Foundations (KAWF) in Sweden, and has maintained a good tradition of updating at least once a year. Currently, HPA has been updating to version 18, which consists of three separate parts, i.e., the Tissue Atlas (TA) [51], the Cell Atlas (CA) [2] and Pathology Atlas (PA) [52]. In this paper, the benchmark dataset has been collected and collated from TA, which mainly focuses on the expression profiles of human genes at the protein level. The images in this sub-database had derived from antibody-based protein analysis by using immunohistochemistry, and covered 15,273 genes (78%) with available antibodies, and involved a total of 44 normal tissues in humans.

The collation and verification of the benchmark dataset are critical to the construction of the predictive model. Hence, a carefully checking task has been carried out on the corresponding benchmark dataset of two published papers [16, 17]. These benchmark datasets derive from published literature in [16, 17], which are respectively single-label dataset and multi-label dataset and has been used in references [15, 40]. The benchmark datasets in [16] based on the early version of HPA database, and the other benchmark datasets proposed by the Xu et al. [17] are collected from the 12 version of HPA database.

The comparison between two reported benchmark datasets and protein subcellular localization annotation on the version 18 of HPA has been summarized in Table 5.

**Table 5:** The change of subcellular locations annotation of benchmark datasets in version 18 of HPA

	The number of proteins.			
	Total	Location Consistently	Location Missing	Location Transfer
Xu et al. [17].	28	8	1	19
Murhpy et al. [16].	16	4	2	10

These update of two reported benchmark datasets about protein subcellular localization annotation on the version 18 of HPA has been summarized in the Table 5. As we are concerned, these datasets can no longer be used as benchmark datasets because the label information in these datasets has been updated by HPA. Furthermore, labels of some protein images are completely different with those of the original dataset. For example, the subcellular localization of Arylsulfatase B protein has been updated from the “lysosome” to the “Golgi apparatus” [2]; the subcellular location of protein HSPA5 belongs “ER” subcellular location in the [2], while its subcellular localizations changes in “Cytosol” in the version 18 of HPA. This is how we are motivated; an updating IHC benchmark dataset is collected and collated based on the latest version of HPA.

**Fig. 6:**
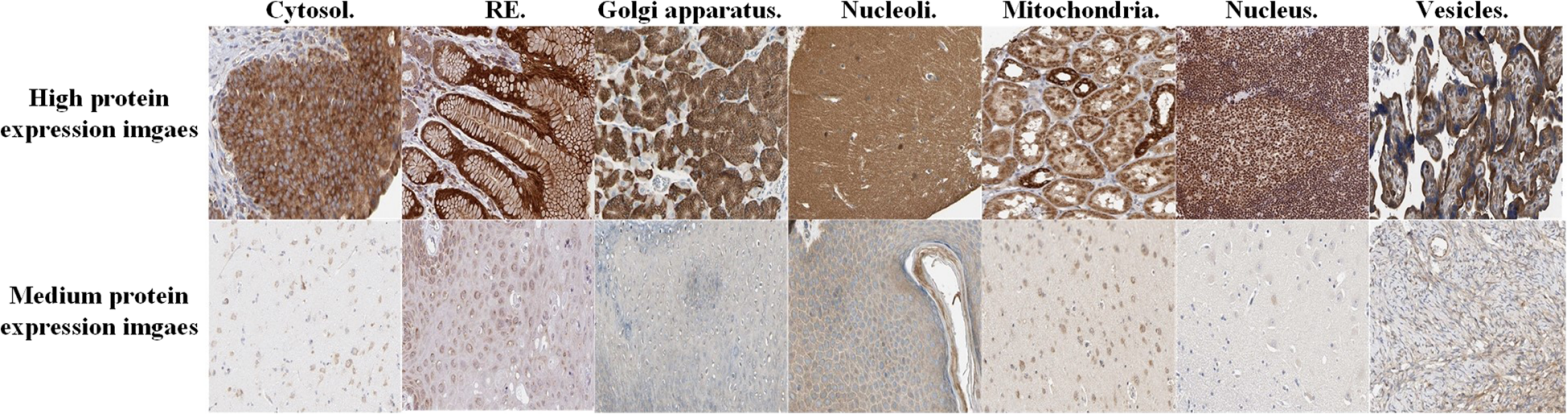
Visual differences of protein images under different subcellular locations and protein expression levels

In addition, each image in HPA has two criterion scores, i.e., reliability score and protein expression level. Both of them play a crucial role in collected a reliable benchmark dataset. The reliability scores are divided into four types, i.e., “Enhanced”, “Supported”, “Approved”, and “Uncertain”. The four types indicate the level of reliability of the analyzed protein expression pattern based on available RNA-seq data, protein or gene characterization data and immunohistochemical data from one or several antibodies with non-overlapping epitopes. For example, the type “Enhanced” is the strictest index among these four reliability score indexes, which not only take the consistency of annotation with other available databases but also utilized the orthogonal or independent antibody validation method. Protein expression level denotes to the protein staining extent of target IHC image, and is divided into four patterns, i.e., “high”, “medium”, “low” and “not detected”. For example, the pattern “high” denotes to the best expression level of protein channel in the target IHC image. To better describe the difference between different protein expression levels, we listed several images with seven subcellular localizations and protein expression levels in Fig. 6.

In this paper, a benchmark image dataset with a total number of 3420 is prepared in consideration of both “Enhanced” and “high” criteria based on version 18 of HPA. The number of proteins with single-label and multi-label are 55 and 25, and the number of images with single-label and multi-label are 2413 and 827. The proportion of protein with multi-label nearly occupies 30%, and the proportion of image with multi-label closes to 25%. The number of the corresponding subcellular organelles is 7, namely “Cytosol”, “Endoplasmic reticulum”, “Golgi apparatus”, “Nucleoli”, “Mitochondria”, “Nucleus”, “Vesicles”. In the process of collecting and collating our benchmark dataset, the same data structure as [17] is followed, namely 70% single-labeled proteins and 30% multi-labeled proteins, which has been listed in Table 6.

**Table 6:** The distribution of protein and image with single-label and multi-label in the benchmark dataset

New Benchmark dataset	Total	Single-label	Multi-label
Proteins	80	55	25
Images	3240	2413	827

### IHC image preprocessing

Different from natural and facial images, the preprocessing of IHC protein images requires a separation of protein channel from original IHC image rather than image rectification or illumination normalization. Each IHC image in HPA contains both DNA and protein components, to which correspond purple and brown color respectively, and photographed by an RGB camera. Hence, the three most important steps in the preprocessing of IHC image can be summarized as follows. Firstly, the transform stage, the original IHC protein image is transformed from RGB space to HSV space, and then filtering at hue level. Secondly, the filtering stage, a certain threshold named dyed index (DI) is employed to filter out badly dyed images, and is fixed at 13 in general [16]. Thirdly, separation stage, linear separated method is employed to achieve precise separation at signal and numerical levels [54].

### Traditional feature

In the field of protein subcellular localization prediction, there are numerous image features regarded as the excellent feature for the IHC image, such as LBP [42], CLBP [44] and SLFs [31]. LBP calculates the gray value of center pixel with the neighboring pixels as statistic information for a target image. CLBP adds coding the property of center pixels on the basis of LBP. The Haralick texture and DNA spatial distribution feature are one of the most discriminative features of SLFs to describe the IHC image from a global perspective, and it has been widely used in many works and has validated its high-performance [15–17, 31, 34, 40, 41]. In this paper, the SLFs feature, derived from the combination of Haralick feature and the DNA distribution feature, is unified into global feature in total 840-dimension [54]. The employment of wavelet transformation has played a positive role in global feature quantization and extraction of IHC images. It has been demonstrated that frequency domain information has certain advantages in describing global feature of IHC images.

However, most research papers prefer to employ an image descriptor to extract features from target protein images in the spatial domain because they only focus on the image properties of digital signals, and ignore the signal properties of its own [55, 35]. Richer information can be observed through signal processing, for example, transforming the target signal from the spatial domain to the frequency domain.

In this paper, frequency feature of IHC image is extracted from these three components of monogenic signal of image based on different frequency scales rather than grey level information, while Haralick features and DNA distribution features being employed to describe the IHC image as the complementary global feature.

### Local image descriptor extraction on frequency domain

Although the conventional features, such as SLFs, LBP, CLBP, can describe the IHC image to some extent. However, local information of IHC image especially in amplitude, phase and orientation aspects are not well mined. In this paper, the target IHC image is transformed into the frequency domain from the spatial domain by the fast fourier transform (FFT). And then, the Riesz transformation is employed to generate the corresponding monogenic signal in frequency domain, which composes three parts i.e., a real part and two imaginary parts. The three parts can be considered as original frequency information and two frequency response parts in signal processing. In order to understand in-depth the protein image signal, Log-Gabor is employed to filter with different frequency scales because it not only inherits the essential property of traditional Gabor filter reflecting the information of specific frequency band in a specific direction but also avoid the influence of DC signal [56]. By using Log-Gabor filter with different frequency scales, local frequency information, which distributes in different frequency bands, can be captured and extracted [57]. Finally, the three parts of different frequency scales are transformed back to the spatial domain respectively.

**Fig. 7:**
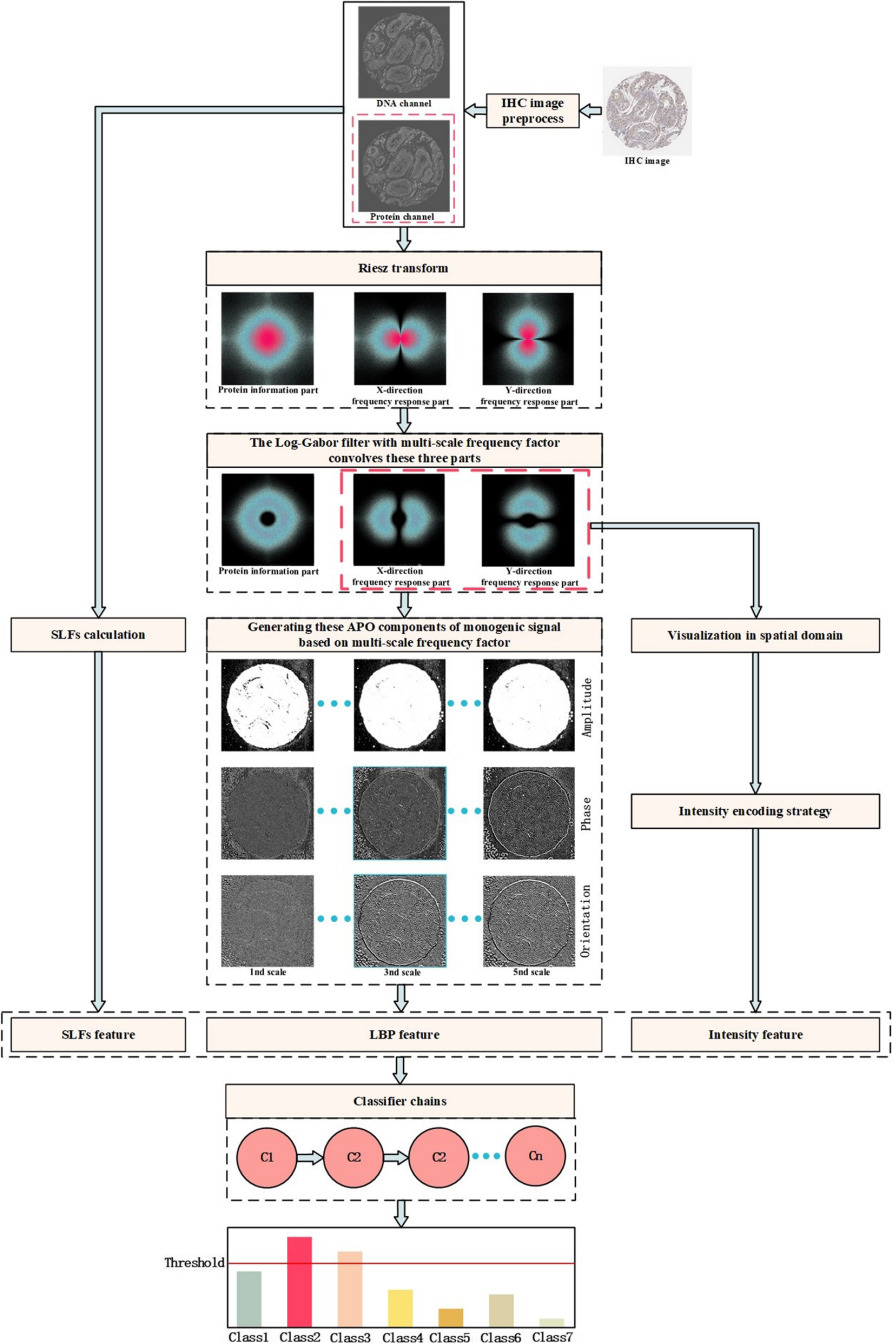
The flowchart of proposed MIC_Locator. The IHC image is selected from gene “ENSG00000013364”. The corresponding number of IHC image is “6980_A_4_6”, and it belongs to the “Cytosol” subcellular location. In the preprocess stage, the DNA and protein channel of protein are separated. On the one hand, the DNA and protein channel are used to extract the 840-dimension SLFs feature. On the other hand, the protein channel is transformed into the frequency domain by the Fourier transform. The frequency information of protein is multiplied by the Riesz transform, generating two frequency responses in orthogonal directions. The frequency information of protein and two frequency response parts of Riesz transform are multiplied by the Log-Gabor filter with multi-scale frequency factor. Afterwards, the protein information and two frequency response parts are transformed into the spatial domain, which commonly consist of the monogenic signal of protein. The APO components of image monogenic signal are calculated. The 8-bits LBP code extracts the statistic information of APO component, and the 2-bits image intensity code is calculated from the two imaginary parts of monogenic signal by the formula (19). The LBP, image intensity and SLFs are united as the final 1864-dimension sample feature, feeding into the CC. The top and threshold criteria are applied to judge the subcellular localizations of test sample

Since the monogenic signal consists of a real part and two imaginary parts, it is numerically unsuitable for feature extraction of the target signal. Hence, some numerical operations have been done on these three parts so that it can provide more information about the original signal, for example, amplitude (A), phase (P) and orientation (O), and the corresponding formula is given by formula (4, 5, 6). The A component can well represent the edge and contour information of each IHC image, and the P component can well represent structural information and the O component can reflect the geometry information. And then, an efficient 8-bit LBP coding strategy is used to extract the statistic features of three components. Besides, these two imaginary parts are compared with a threshold 0, and generating the 2-bits binary code is considered as the image intensity code. Finally, the image intensity coding and LBP are combined as the 1024-dimension local image descriptor. The Haralick feature united the local image descriptor as a sample feature of 1864-dimension, feeding into CC to construct the prediction model. The details of local image descriptor coding have been described in the next section. Finally, the average and weighted ensemble method are employed to fuse the probability scores at prediction level. The top and threshold criteria are proposed to give the final decision of subcellular locations. The flowchart of proposed MIC_Locator is shown in Fig. 7. The meaning of the proposed prediction model, MIC_Locator, can be summarized as follows: letter “M” denotes to monogenic signal; letter “I” denotes to image intensity coding strategy; letter “C” represents to classifier chain; word “Locator” stands for the goal of subcellular localization.

### APO components generation of monogenic signal

Frequency domain signal analysis (FDSA), as one of the most important approaches in the field of signal processing, can show in depth how many sub-signals lie within each given frequency band over a range of frequencies, and these different frequencies can well represent approximate information and detailed information of the original signal. At the level of mathematical analysis, the primary purpose of FDSA is to obtain the analytic signal of target signal, for example, the combination of a 2-D signal with the Riesz transformed one yields a sophisticated 2-D analytic signal. The analytic signal approach was introduced by Felsberg M, Sommer G in 2001 [46] and has been widely applied to many fields, such as medical image analysis [58] and synthetic-aperture radar (SAR) image recognition [59].

In this paper, Riesz transform, defined as a high-dimension generalization of the Hilbert transform, is employed to transform the original signal into a new signal on a 2-D complex plane. In 2-D plane, the Riesz transform can be expressed as follow.

1 $$ {S}_R{(p)}_{x,y}=\left(\begin{array}{c}{S}_x(p)\\ {}{S}_y(p)\end{array}\right)=\left(\begin{array}{c}{h}_x\ast s(p)\\ {}{h}_y\ast s(p)\end{array}\right) $$

where *s*(*p*) denotes to the original or target signal. X and Y are the two orthogonal directions of the 2-D complex plane, and the entire 2-D Hilbert space has been spanned by Riesz transform. *h*_*x*_ and *h*_*y*_ is defined as Hilbert transform factor, and the corresponding Fourier transform can be defined as *H*_*x*_ =  − *jw*_*x*_/‖*ω*‖ and *H*_*y*_ =  − *jw*_*y*_/‖*ω*‖ with the angular frequency *ω* = (*ω*_*x*_, *ω*_*y*_). The character *R* of *S*_*R*_(*p*)_*x*, *y*_ symbolizes the Riesz transform or 2-D Hilbert transform of image. The Riesz transform kernel is defined as follow.

2 $$ \left({h}_x,{h}_y\right)=\left(\frac{x}{2\pi {\left\Vert p\right\Vert}^3},\frac{y}{2\pi {\left\Vert p\right\Vert}^3}\right) $$

Thus, for target signal *s*(*p*), the corresponding monogenic signal is defined as follow:

3 $$ {S}_M{(P)}_{x,y}=\left(S(p),{S}_x(p),{S}_y(p)\right) $$

where *S*(*p*) denotes to the real part of the monogenic signal. *S*_*x*_(*p*) and *S*_*y*_(*p*) are the two imaginary parts along the X-axis and Y-axis direction respectively. Finally, the APO components can be obtained by using formula (4, 5, 6).

4 $$ A=\sqrt{S^2+{S}_x^2+{S}_y^2} $$

5 $$ \phi =\mathrm{atan}2\left(\sqrt{S_y^2+{S}_x^2}/S\right) $$

6 $$ \theta =\mathrm{atan}2\left({S}_x/{S}_y\right) $$

The function atan(x/y) presents the arctan(x/y) function, and the value range of the function atan(x/y) arranges [−*pi*/2, *pi*/2] and covers two quadrants. In contrast, the value range of function atan2(*x*, *y*) is [−*pi*, *pi*] covering four quadrants, and the value of element in these PO components same belongs [−*pi*, *pi*]. Hence, the function atan2(*x*, *y*) is employed to calculate the value of element these PO components. Where A denotes to amplitude (A) component, and *ϕ* denotes to phase (P) component, and *θ* denotes to orientation (O) component.

### Multi-scale monogenic signal representation

It is well known that the representation of target signal in frequency domain is much more explicit than spatial domain because the energy of target signal is more concentrated in frequency domain. Furthermore, this is benefited by the multi-scale decomposition of target signal in frequency domain. For example, the interested region of image in spatial domain, such as patches consisting of contour or edge information, can be easily captured and represented in the frequency domain. Inspired by this, the Log-Gabor filter with the logarithmic mapping function is employed to achieve multi-scale decomposition in this paper. The advantage of the Log-Gabor filter is a more desirable frequency response especially in the high-frequency band while comparing with the traditional Gabor filter [57]. Moreover, the Log-Gabor filter can avoid the influence of DC, which limits the bandwidth of band-pass filter. The definition of the Log-Gabor filter is shown as follow.

7 $$ G\left(\omega \right)=\exp \left\{-{\left[\mathrm{Log}\left(\omega /{\omega}_0\right)\right]}^2/2{\left[\mathrm{Log}\left(\sigma /{\omega}_0\right)\right]}^2\right\} $$

8 $$ {\omega}_0={\left(\lambda {k}^{r-1}\right)}^{-1} $$

where *ω*_0_ denotes to the center frequency. The *λ* is defined as the setting minimum wavelength, and it is set 4. The *k* is the multiply factor of wavelength, which equals 1.7. The *σ*/*ω*_0_ is set as a constant value to make the Log-Gabor with a constant shape ratio, which is set 0.64. The *r* is the scale index, and its intervals are from 1 to 5. The parameters are set according to the recommendation in [47] and our own experiments result.

**Fig. 8:**
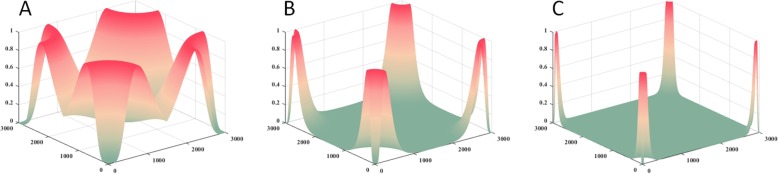
The frequency response of Log-Gabor filter with different frequency scale factors. **a**, **b** and **c** Respectively present the frequency response of Log-Gabor filter based on the frequency scale factor 1, 3 and 5

With changing the frequency scale factors from 1 to 5, the frequency response of Log-Gabor filter has been shown in Fig. 8. Specifically, the center region is caved in the frequency response of Log-Gabor filter. The phenomenon denotes to the current direct by avoided, and the low frequency information can be restrained. Meanwhile, with the frequency scale increase, the frequency response of Log-Gabor filter in high frequency band can be apparently improved.

Then, the band-pass monogenic signal is obtained by making the convolution of original signal and Log-Gabor, which has been shown in the formula (9).

9 $$ {S}_{LG-M}\left(\mathrm{p}\right)=\left({S}_{LG}(p),{S}_{LG-\mathrm{x}}(p),{S}_{LG-y}(p)\right)=\left({S}_{LG}(p),{h}_x\ast {S}_{LG}(p),{h}_y\ast {S}_{LG}(p)\right) $$

10 $$ {S}_{LG}(p)=S(p)\ast {F}^{- 1}\left(G\left(\omega \right)\right) $$

11 $$ {S}_{LG-x}(p)={h}_x\ast {S}_{LG}(p) $$

12 $$ {S}_{LG-y}(p)={h}_y\ast {S}_{LG}(p) $$

In formula (10), the *F*^−*1*^ denotes to the 2D inverse Fourier transform, and *S*_*LG*_(*p*) is the real part of monogenic signal convolving the Log-Gabor filter. The *S*_*LG* ‐ *x*_(*p*) is the X-direction imaginary part of monogenic signal convolving the Log-Gabor filter in formula (11), and *S*_*LG* − *y*_(*p*) is the Y-direction imaginary part of monogenic signal convolving the Log-Gabor filter in formula (12). The corresponding APO components are updated as follows.

13 $$ {A}_{LG}=\sqrt{S_{LG}^2+{S}_{LG-x}^2+{S}_{LG-y}^2} $$

14 $$ {\phi}_{LG}=\mathrm{atan}2\left(\sqrt{S_{LG-y}^2+{S}_{LG-x}^2}/{S}_{LG}\right) $$

15 $$ {\theta}_{LG}=\mathrm{atan}2\left({S}_{LG-x}/{S}_{LG-y}\right) $$

To represent intuitively, APO components under different scales have been shown in Fig. 7. For A component, it reflects the shape of an image and describes local energetic information. For local phase and orientation component, these two components denote to local structure and geometry information.

### Monogenic signal encoding and feature quantification

An effective encoding method is not only the accurate quantification of the target signal but also can provide more discriminative features to the subsequent classifiers. In this paper, two encoding strategies, i.e., general encoding strategy and intensity encoding strategy, are employed to quantify target IHC image. The former strategy encodes APO components, i.e., *A*_*LG*_ *ϕ*_*LG*_ and *θ*_*LG*_, by using traditional LBP encoding method, which calculates the relationship between the center pixel and its surrounding pixels in the target local region. The latter strategy focuses on encoding the variation consistency of two imaginary parts of monogenic signal. Obviously, these two encoding strategies work on the local region of target image, and then perform statistics and quantization. The processing of monogenic signal generation has been shown in Fig. 7, and the details of LBP descriptor can be found in [42].

### General encoding strategy of APO components

The traditional LBP encoding strategy has been widely applied in many fields related to image processing, such as cell localization and phenotype recognition due to its simple and efficient characteristics [60, 61]. The corresponding formula is given below.

16 $$ {K}^{N,r}\left({p}_c\right)=\sum \limits_{i=1}^N{2}^{\left(\mathrm{i}-1\right)}\ast L\left({p}_i-{p}_c\right),\kern1em L(x)=\left\{\begin{array}{cc}1,& x\ge 0\\ {}0,& else\end{array}\right. $$

where *p*_*c*_ stands for the center pixel in each local region, and *p*_*i*_ denotes to a neighboring pixel. *N* represents the number of neighboring pixels, and *r* denotes to the radius of neighborhood. *L*(*x*) is a symbol function, and the function value is defined as 0 when independent variable is negative. The *K*^*N*, *r*^(*p*_*c*_) presents the LBP coding of each center pixel in spatial domain.

To extract the statistic information of local amplitude, the local amplitude component is normalized to [*0*, *255*]. However, local orientation and local phase components represent an angle with a specific direction, and the corresponding value is ranged from [−*pi*, *pi*], which is unlike with that of local amplitude component. Hence, P and O components are required special numerical coding. The general encoding strategy of APO components can be summarized as follows.

#### The encoding strategy of local amplitude component

**Fig. 9:**
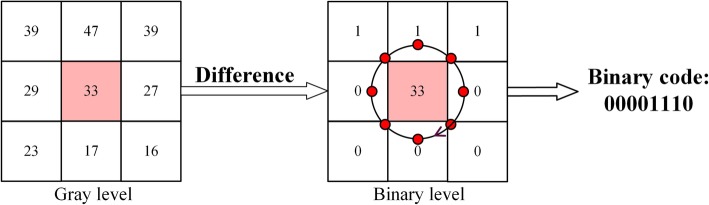
The LBP coding process of amplitude component in a local patch. The starting point of the LBP coding is in the lower right corner and encoded in a clockwise direction

The local amplitude component represents the energetic information of local region in target IHC image. Hence, taking into account the property of amplitude component, and the interval of local amplitude is normalized to [*0*, *255*]. The standard encoding strategy of LBP is employed to quantize amplitude component feature. In detail, if the grey level of neighbor pixels is larger than the center pixel, and then the value of neighbor pixels is encoded as 1; whereas, the value of neighbor pixels is encoded as 0 if the grey level of neighbor pixels is smaller than the grey level of center pixel. The coding process of amplitude component has been shown in Fig. 9.

#### The encoding strategy of local phase and orientation components

Different from amplitude component in the monogenic signal, the elements of phase and orientation component range in value from [−*pi*, *pi*]. Considering the physical meaning of local orientation and local phase, namely, the different value of local orientation and the local phase is associated with the corresponding types of feature. For example, two phases are close to 0, which presents that feature type of two elements is similar and belongs step edge; two orientations are close, and it means that the gradient direction of two elements are almost along a same direction.

Therefore, a quadrant encoding strategy is employed in this study. In detail, each element of local orientation and phase component is normalized to [*0*, *359*]. Then, we divided the range of [*0*, *359*] into M intervals (M = 4 while set quadrant encoding), i.e., [*0*, *89*), [*90*, *179*), [*180*, *269*) and [*270*, *359*), and the corresponding value falling in each interval is encoded as “0”, “1”, “2” and “3” respectively.

Obviously, each quadrant coding is different from others, and related to different types of feature described in [47], for example, different phase angles. The coding formulas of the local phase and orientation component are given as follows.

17 $$ {X}_i\left({p}_c\right)=\left\{\begin{array}{cc}0&\ if\ Q\left(\Phi \left({p}_c\right)\right)=Q\left(\Phi \left({p}_i\right)\right)\\ {}1& else\end{array}\right. $$

18 $$ Q(Deg)=p,\kern0.5em if\ \frac{360\cdot \left(p-1\right)}{M}\le Deg<\frac{360\cdot p}{M}\kern0.5em $$

**Fig. 10:**
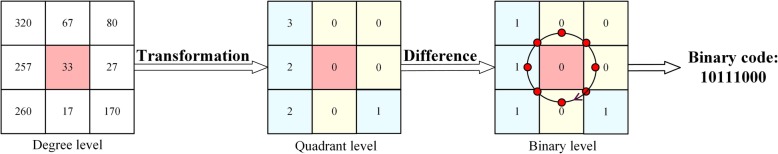
An example of encoding phase and orientation components of monogenic signal. The value of phase and orientation component is converted into four intervals, and four intervals present different types of feature. Afterwards, the LBP of phase and orientation components is generated, and LBP code begins to generate from the bottom right corner in clockwise direction

For the orientation and phase components, Φ(*p*_*c*_) represents the value of each center pixel *p*_*c*_, and Φ(*p*_*i*_) represents the value of neighboring pixel *p*_*i*_. Meanwhile, the formula (18) is the quantification of local phase and orientation. The coding process of phase and orientation component has been shown in Fig. 10.

### Image intensity encoding strategy

Inspired by the characteristics of CLBP feature [44], taking the property of center pixel into account, an encoding strategy named intensity encoding is proposed to generate a complementary feature coding for LBP coding of APO components.

**Fig. 11:**
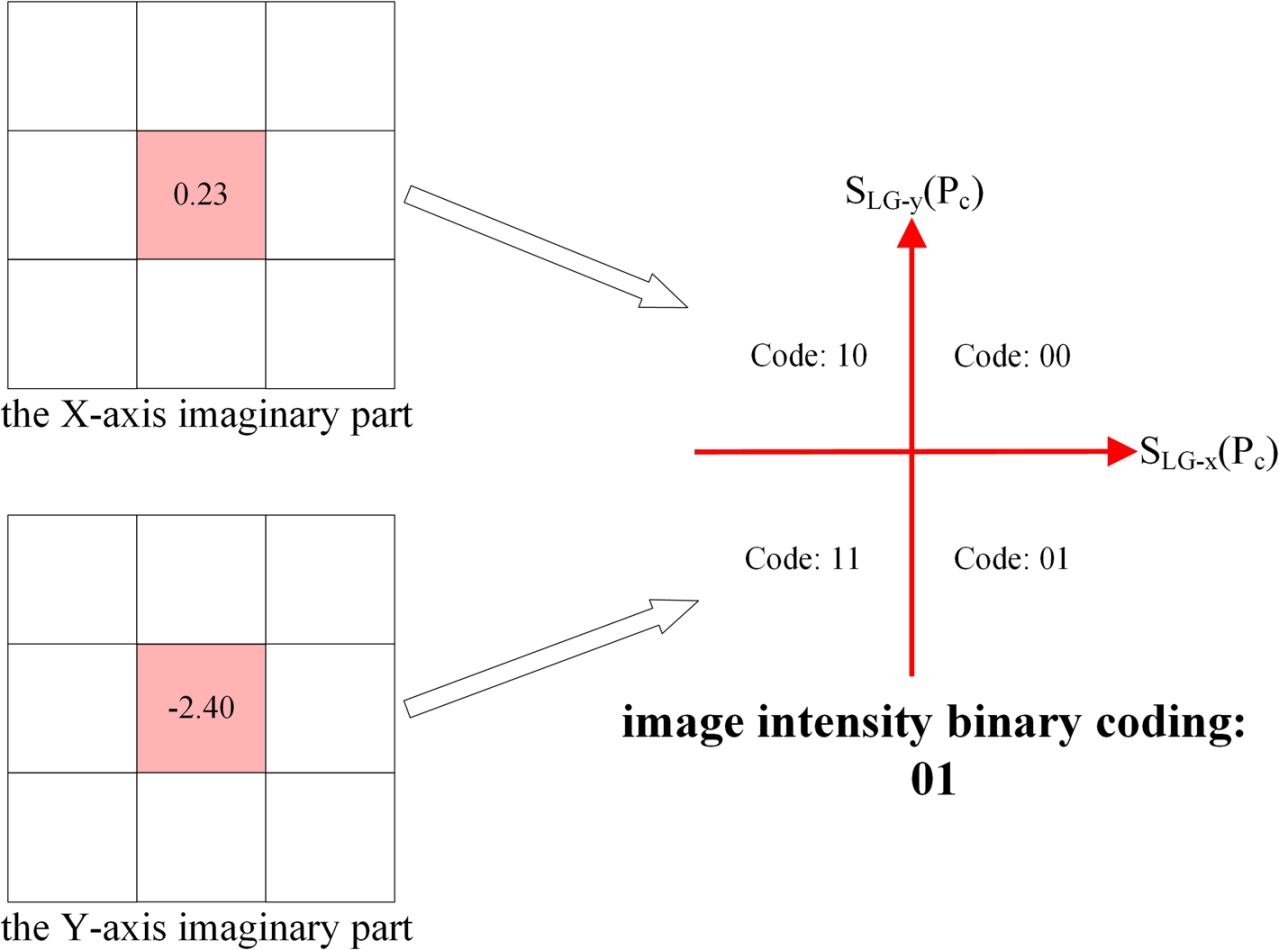
The image intensity coding process of center pixel in frequency domain. The two imaginary parts of monogenic signal in the X and Y direction are compared to the threshold value 0. The comparison result is mapped into the four quadrants, and four quadrants respectively stand for four 2-bits codes, “00”, “10”, “11” and “01”, as the image intensity code. As the value of X-direction and Y-direction imaginary part are 0.24 and − 2.4, the image intensity binary code of element is “01”

The two imaginary parts originated from the monogenic signal of protein channel can be considered as the representation of each target IHC image in 2-D Hilbert space. Hence, the variation consistency of two imaginary parts of monogenic signal is captured and encoded as a 2-bits code corresponding to 4 patterns, which has been shown as follow.

19 $$ \left[{C}_x^I\left({p}_c\right),{C}_y^I\left({p}_c\right)\right]=\left\{\begin{array}{cc}00& if\kern0.5em {S}_{LG-x}\left({p}_c\right)>0\ \mathrm{and}\ {S}_{LG-y}\left({p}_c\right)>0\\ {}10& if\kern0.5em {S}_{LG-x}\left({p}_c\right)<0\ \mathrm{and}\kern0.5em {S}_{LG-y}\left({p}_c\right)>0\\ {}11& if\kern0.5em {S}_{LG-x}\left({p}_c\right)<0\ \mathrm{and}\ {S}_{LG-y}\left({p}_c\right)<0\\ {}01& if\kern0.5em {S}_{LG-x}\left({p}_c\right)>0\ \mathrm{and}\ {S}_{LG-y}\left({p}_c\right)<0\end{array}\right. $$

where *S*_*LG* − *x*_ and *S*_*LG* − *y*_ (refer to formula 9) please) are the two imaginary parts of monogenic signal. Comparing these two imaginary parts of monogenic signal with the threshold 0, the 2-bits image intensity code can be generated, “00”, “10”, “11” and “01”, and the process of image intensity coding have been shown in Fig. 11.

### The qualitative analysis of image intensity encoding strategy

**Fig. 12:**
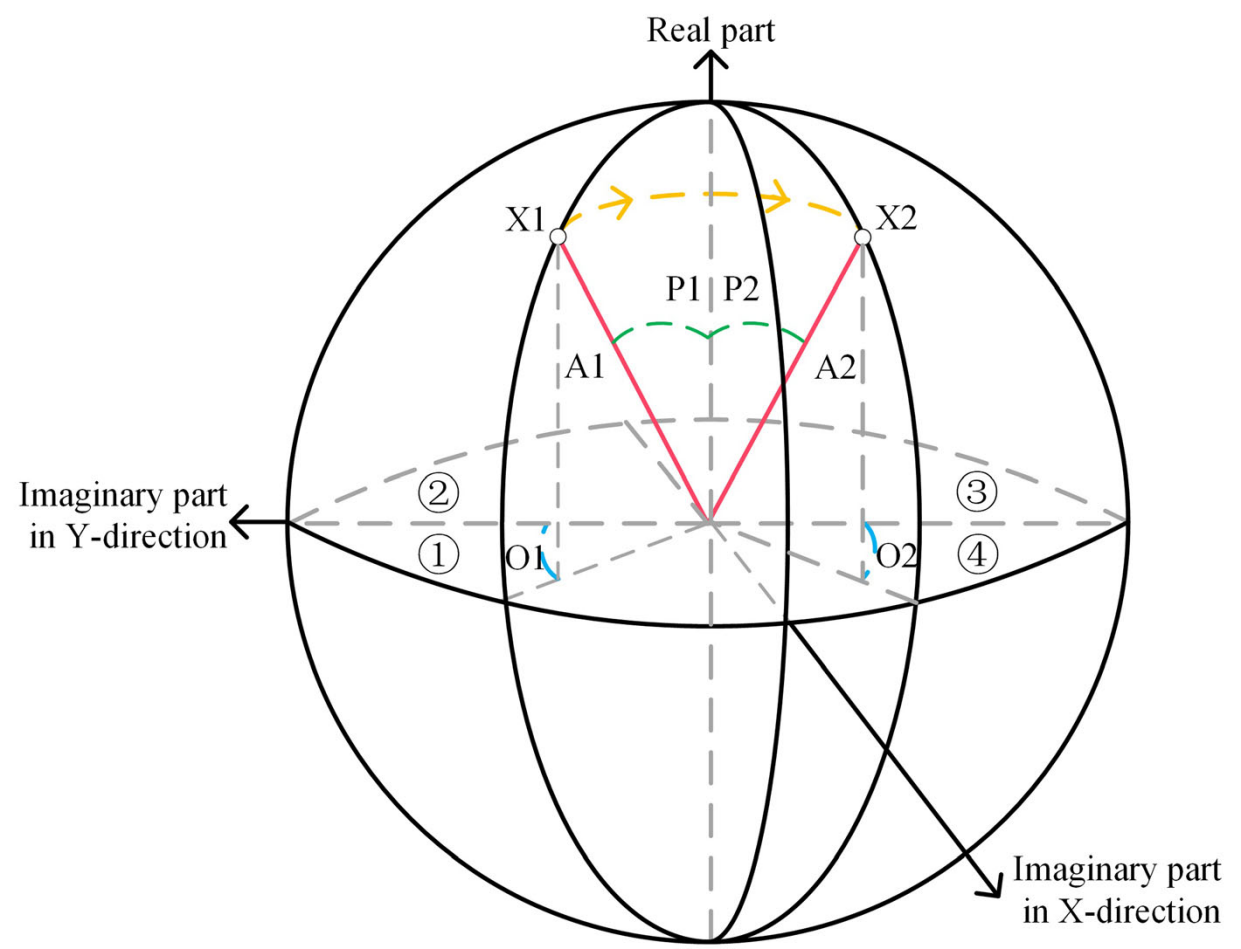
The spherical coordinate system representation of monogenic signal. The z-axis is the real part of monogenic signal. The X-axis and Y-axis are respectively the two imaginary parts of monogenic signal in the X and Y direction. In the Spherical coordinate system, these are four regions dividing into 4 regions according to the formula (19). The X1 is a sample in region 1, and its amplitude, phase and orientation are A1, P1 and O1 which are respectively marked by the red, green and blue. The X2 is generated by rotating the X1 90 degree in an anti-clockwise direction located in region 4, and the rotation direction is presented by the yellow color. These amplitude, phase and orientation components of X2 are A2, P2 and O2, where A2, P2 and O2 components are respectively marked by the red, green and blue

The characteristics of Hilbert transformation is phase shift 90 degree based on the original signal, and the Riesz transform consists of two Hilbert transform in X and Y directions. Hence, the monogenic signal can be presented in a spherical coordinate system. These two imaginary parts of monogenic signal along the X and Y direction can be regarded as the X-axis and Y-axis of spherical coordinate system, and the Z-axis is equal to the real part of monogenic signal. The spherical coordinate system representation of monogenic signal has been shown in Fig. 12. Samples contribute in the surface of spherical coordinate system, and these components of monogenic signal can be calculated. For instance, a given sample X1, the amplitude component of X1 is the distance of X1 and the origin, which is presented as the A1 and is remarked by the red. The phase component is an angle between the Z-axis and the amplitude component A1, which is P1 and remarked by the green color. The orientation component of sample is an angle between the imaginary part in Y-direction and the projection of A1 in the XY plane, such as O1 which belongs to the orientation components of X1 and remarked by the blue color. Supposing the sample X2 is generated by rotating the sample X1 with 90 degree in the anticlockwise, and the rotation is remarked by the yellow color. Then the three components of sample X2 are generated, A2, P2 and O2. It is considerably obvious that values of A2 and P2 are same as these A1 and P1, and the O2 and O1 are various. The similar APO components value of sample easily leads the prediction model lacking the discriminative and generation ability. The key problem is how to distinguish these similar samples in the entirely spherical system, such as X1 and X2.

In this study, the spherical system is divided into four regions. The X-axis and Y-axis of spherical coordinate system is the X-direction and Y-direction of imaginary part of monogenic signal. By the formula (19), these four regions respectively response to these four image intensity codes, “00”, “01”, “11” and “01”. By coding the image intensity, X1 and X2 can be distinguished. Such as the X1 in the region 1 and the X2 in the region 4, and the image intensity code respectively is “00” and “01”. The 2-bits image intensity code is concatenated on 8-bit LBP as a final 10-bit local image descriptor.

### Chains classification and fusing strategy of prediction model

As the aforementioned, the local image descriptor consists of the LBP code in these three APO components and image intensity code, and the 1864-dimension sample feature is formed by combining the local image descriptor and global image feature (SLFs features). The stepwise discriminant analysis (SDA) feature selection method is used to select the discriminative feature subset from the original feature space, which uses the Wilks’ λ statistic to iteratively judge which features are the most discriminating. The selected feature subset is fed into the CC. Considering the correlation of labels in the multi-label datasets, the classifier chain approach is employed to handle multi-label datasets classification. The CC consists of several binary SVM classifications, and the probability score of previous SVM outputs is added into the feature space in the next SVM classification so that CC can capture the correlation of label.

Under the different APO components and the frequency scales factors of Log-Gabor, constructing the prediction model is presented MIC_Locator^X_S (^the x is A, P and O components; S denotes to the frequency scale factor Log-Gabor from 1 to 5). Because prediction model with the various frequency scale factor S, namely MIC_Locator^A_1^, MIC_Locator^A_2^, MIC_Locator^A_3^, MIC_Locator^A_4^ and MIC_Locator^A_5^, has various discriminative for information distributing in different frequency bands, the average ensemble approach is used to sum the seven prediction probability scores of MIC_Locator^X_S^ in each component. The MIC_Locator^X_E^ is an ensemble prediction model based on three components, and X denotes to amplitude, phase or orientation components.

Finally, we summed the probabilities value deriving from the three ensemble prediction models of monogenic components. As the amplitude, phase and orientation component of monogenic signal mainly reflects the local energetic information, the local structural and the local geometric information along main orientation respectively, and the phase and orientation components can describe the image texture superior to the amplitude component. The weighted ensemble algorithm is applied to fuse these three prediction models based on the APO components. The formula of weighted ensemble algorithm has been shown as follow:

20 $$ {S}_{FDI\_ PSL}=\left(1-2\ast w\right)\ast {S}_{\mathrm{MIC}\_{Locator}^{A\_E}}+w\ast {S}_{\mathrm{MIC}\_{Locator}^{P\_E}}+w\ast {S}_{\mathrm{MIC}\_{Locator}^{O\_E}} $$

where *W* is the weight and is set 0.43. The extensive experiment of selecting *W* has been shown in Fig. 12 and in the next section. By the formula (20), we can build the MIC_Locator prediction model. Refer to all 10 vanishing moments, we summed the prediction probabilities of test images of prediction model output and divided the sum value by the number of 10 vanishing moments.

## Data Availability

The selected benchmark dataset can be available in the website (https://github.com/ProteinLocator/MIC_Locator) for the academic research.

## Abbreviations

AAC: Amino acid composition
APO: Amplitude, phase and orientation
BR: Binary relevance classifier
CA: Cell atlas
CC: Multi-label classifier chains
CDD: Conserved domain database
CLBP: Completed local binary pattern
CNN: Convolution neural network
DC: Direct current
DI: Dyed index
ECOC: Error-correcting output codes strategy
FDSA: Frequency domain signal analysis
FFT: The fast fourier transform
GO: Gene ontology
GPCR: G Protein-Coupled Receptor
HPA: Human protein atlas database
IHC: Immunohistochemistry
KAWF: The Knut and Alice Wallenberg Foundations
KNN: K-nearest neighbor classifier
LBP: Local binary pattern
LQP: Local quinary pattern
LTP: Local ternary pattern
LTrP: Local tetra pattern
PA: Pathology atlas
PSSM: Position specific scoring matrix
RALS: Random label selection method
SDA: Stepwise discriminant analysis
SLFs: Subcellular location features
SVM: Support vector machine
TA: Tissue atlas
